# Immunohistochemical analysis of p53 in vulval intraepithelial neoplasia and vulval squamous cell carcinoma

**DOI:** 10.1038/sj.bjc.6600677

**Published:** 2003-01-28

**Authors:** A N Rosenthal, D Hopster, A Ryan, I J Jacobs

**Affiliations:** 1 Department of Obstetrics and Gynaecology, The Gynaecological Oncology Unit, Bart's and The London, Queen Mary School of Medicine and Dentistry, London University, Charterhouse Square, London EC1M 6GR, UK; 2 Academic Department of Histopathology, Bart's and The London, Queen Mary School of Medicine and Dentistry, London University, West Smithfield, London EC1A 7BE, UK

**Keywords:** LOH, P53, HPV, VIN, vulval cancer

## Abstract

Human papillomavirus (HPV) is thought to cause some vulval squamous cell carcinomas (VSCC) by degrading p53 product. Evidence on whether HPV-negative VSCC results from p53 mutation is conflicting. We performed immunohistochemistry for p53 product on 52 cases of lone vulval intraepithelial neoplasia (VIN), 21 cases of VIN with concurrent VSCC and 67 cases of VSCC. We had previously performed HPV detection and loss of heterozygosity (LOH) analyses on these samples. Abnormal p53 immunoreactivity (p53-positive) rates in HPV-positive VSCC and HPV-negative VSCC were 22% (12/54) and 31% (4/13), respectively (*P*<0.74). p53 immunoreactivity was associated with LOH at the p53 locus (*P*<0.004), but neither technique differentiated between HPV-positive and HPV-negative VSCC. p53 immunoreactivity was associated with overall LOH rates (p53-positive VSCC *vs* p53-negative VSCC mean fractional regional allelic loss 0.41 *vs* 0.24, respectively, *P*<0.027). LOH at 3p25 was more frequent in p53-positive VSCC cf p53-negative VSCC (70 *vs* 21%, respectively, *P*<0.007). There was a trend in p53 disruption associated with invasive disease; HPV-positive VSCC demonstrated more disruption than VIN associated with VSCC, which had more disruption than lone VIN III (22 *vs* 10 *vs* 0%, respectively, *P*<0.005). In all, three out of 73 cases of VIN were p53-positive. All three were associated with concurrent or previous VSCC. Meta-analysis of previous studies revealed significantly more p53 disruption in HPV-negative VSCC cf HPV-positive VSCC (58 *vs* 33%, respectively; *P*<0.0001). p53 immunoreactivity/mutation in VIN only appeared in association with VSCC. These data suggest that HPV-independent vulval carcinogenesis does not exclusively require disruption of p53, p53 disruption may work synergistically with LOH at specific loci and p53-positive VIN should be checked carefully for the presence of occult invasion.

The p53 tumour suppressor gene is central to the development of many solid tumours. It plays key roles in cell cycle regulation and apoptosis. Oncogenic human papillomavirus (HPV) types are thought to cause cervical squamous cell carcinoma. The oncogenic HPV gene products E6 and E7 act on p53 and Rb, respectively. E6 targets p53 product for degradation via the ubiquitin pathway (Scheffner *et al*, 1990), while E7 complexes and inactivates Rb (Dyson *et al*, 1989). A proportion of vulval squamous cell carcinoma (VSCC) and the vast majority of vulval intraepithelial neoplasia (VIN) are associated with oncogenic HPV infection (Hording *et al*, 1994; Kohlberger *et al*, 1998; Rosenthal *et al*, 2001). The different patients' ages (Hording *et al*, 1994; Monk *et al*, 1995), histological subtypes (Hording *et al*, 1994; Monk *et al*, 1995) and the presence of VIN (Hording *et al*, 1994; Ngan *et al*, 1999) in HPV-positive but not HPV-negative VSCC, all support the hypothesis that VSCC can arise via both an HPV-dependent and HPV-independent pathway. Few studies have addressed potential differences in the molecular events in these two groups. Loss of heterozygosity (LOH) data from ourselves (Rosenthal *et al*, 2001) and others (Flowers *et al*, 1999; Pinto *et al*, 1999) suggest that HPV-positive and HPV-negative VSCC consistently undergo loss of different chromosomal loci. In addition, another study found differing DNA ploidy in HPV-positive and HPV-negative VSCC (Scurry *et al*, 1999).

We hypothesised that as HPV-positive VSCC was likely to involve p53 dysfunction because of viral E6 gene product, HPV-negative VSCC might also involve abrogation of the function of this tumour suppressor gene. Data on p53 in this context are conflicting. While some studies suggest that p53 mutation is absent (Kurvinen *et al*, 1993; Kim *et al*, 1996) or rare (Lee *et al*, 1994) in HPV-positive VSCC, but present in about one-third of HPV-negative VSCC, other studies, using a variety of techniques, including immunohistochemistry (IHC), have found 31–48% of HPV-positive VSCC and 58–75% of HPV-negative VSCC to show aberrant p53 expression and/or mutation (Milde-Langosch *et al*, 1995; Pilotti *et al*, 1995; Kagie *et al*, 1997a; Flowers *et al*, 1999; Ngan *et al*, 1999). Although all these studies show a trend towards higher proportions of HPV-negative VSCC exhibiting p53 dysfunction compared with HPV-positive samples, none of these studies reached statistical significance. We therefore wanted to try to clarify this issue in a large series and perform a meta-analysis of all these series.

We also wanted to investigate p53 immunoreactivity as a marker of progression to invasion. Some studies suggest absent p53 immunoreactivity in lone VIN (Kurvinen *et al*, 1993; Tervahauta *et al*, 1993; Pilotti *et al*, 1995; Kohlberger *et al*, 1998), while others have found 17–52% p53 immunoreactivity in VIN associated with VSCC (Milde-Langosch *et al*, 1995; Kagie *et al*, 1997a,1997b; McConnell *et al*, 1997; Emanuels *et al*, 1999). It is therefore possible that p53 immunoreactivity could represent a marker for risk of invasion. Higher rates of p53 immunoreactivity in VIN from patients with VSCC compared to those without could indicate a role for this gene in progression from VIN to VSCC. Finally, because we had already investigated our samples for LOH at six different chromosomal loci (Rosenthal *et al*, 2001), it was possible to examine any relation between aberrant p53 immunoreactivity and LOH at specific loci. Here we report the first study of p53 IHC in a large series of VIN and VSCC samples, also tested for HPV infection and LOH at multiple chromosomal loci.

## MATERIALS AND METHODS

### Samples

Patients with VIN and VSCC diagnosed between 1989 and 1997 were identified using the computerised database of the pathology departments of St Bartholomew's and the Royal London Hospitals. Samples obtained were as follows: 43 cases of lone VIN III, four cases of lone VIN II, five cases of lone VIN I, 49 cases of VSCC and 21 cases of VIN associated with concurrent VSCC. In all, 18 of the 21 cases of VIN associated with concurrent cancer had the VSCC still remaining on the specimen blocks after serial sectioning. There were therefore 67 cases of VSCC available for study (18 cases associated with concurrent VIN and 49 cases not associated with VIN). Of the 21 cases of VIN associated with VSCC, 18 were VIN III, two were VIN II and one was VIN I. Of the 67 VSCC cases, 24 were stage I, 11 were stage II, nine were stage III, three were stage IV, and in 20 information for accurate staging was not available because specimens were biopsies only. The relevant paraffin-embedded tissue samples were serially sectioned as follows: one 4-*μ*m section was mounted, stained with haematoxylin and eosin, covered and used as a reference slide. One 4-*μ*m section was used for p53 IHC.

### LOH analyses and HPV DNA detection and typing

The methods used for LOH analysis and for detection and typing of HPV DNA in the samples in this study have been previously described in detail (Rosenthal *et al*, 2001). Briefly, DNA was extracted from microdissected archival normal and neoplastic vulval tissue. LOH analysis was performed by PCR using microsatellite markers for the following loci: 17p13–p53, 9p21–p16, 3p25, 4q21, 5p14 and 11p15. HPV DNA was detected by PCR, using highly sensitive consensus genital-type HPV L1 gene primers GP5+ and GP6+ (Kohlberger *et al*, 1998). PCR products were run on agarose gels, and amplification bands from samples positive for HPV DNA were cut out of the gel, and the DNA extracted and sequenced. The resulting sequences were compared with known HPV types using a BLAST search.

### IHC

Samples were dewaxed in three changes of xylene and rehydrated in three changes of methanol (100, 90 and 70%). Following washing in two changes of distilled water, endogenous peroxidase activity was blocked in 3% H_2_O_2_ for 10 min. The samples were washed in two changes of Tris-buffered saline (TBS). Nonspecific binding was blocked using 1 : 10 normal goat serum (Dako, Ely, UK) in phosphate-buffered saline and 0.1% bovine serum albumin (PBS–BSA) for 20 min in a humidity chamber. Slides were incubated overnight in 1 : 50 DO7 anti-p53 mouse monoclonal antibody (Dako, Ely, UK) in PBS–BSA. This step was omitted for negative controls. The samples were then incubated for 45 min in 1 : 100 goat anti-mouse secondary biotinylated antibody (Dako, Ely, UK) in PBS–BSA with 10% normal human serum. The samples were washed in two changes of TBS and incubated for 45 min in 1 : 200 streptavidin–biotin complex (Dako, Ely, UK) in TBS. The samples were washed in two changes of TBS. Staining of primary antibody was performed using the chromagen 3-diaminobenzidine tetrahydrochloride (DAB) (180 mg DAB in 270 ml H_2_O, 30 ml TBS, 1 ml 0.1 M imidazole and 120 *μ*l 30% H_2_O_2_). The slides were counterstained using Mayers Haemalum (Merck, Poole, UK), washed in water, placed briefly in 1% acid-alcohol followed by blueing solution (0.5% disodium tetraborate) and dehydrated in two changes of ethanol. The samples were placed briefly in xylene to restore refractive index and mounted using DPX (Merck, Poole, UK). We used a breast cancer sample known to express p53 as a positive control.

### Assessment of p53 staining pattern

Samples were scored as negative (<10% of nuclei positive), positive (10–50% of nuclei positive) or highly positive (>50% of nuclei positive) (Kagie *et al*, 1997b). These percentages were grossly assessed by counting nuclei. All slides were assessed by two observers (ANR, DH) independently.

### Meta-analyses of previous studies of p53 in vulval neoplasia

Studies were identified using the search terms vulva, vulval neoplasia, VIN and p53 on PUBMED. References from the publications retrieved were also obtained. Since a wide variety of techniques for assessing p53 dysfunction have been used, and not all studies assessed HPV status, we have made the following assumptions in combining these results for statistical analysis. Firstly, all VIN was assumed to be HPV-positive, irrespective of whether HPV detection was performed. This assumption is justified by our own data (Rosenthal *et al*, 2001) and that of others (Hording *et al*, 1995; Kohlberger *et al*, 1998), which indicates that >90% of VIN is HPV-positive. Secondly, >‘moderate staining’ or >‘10% of nuclei staining positive’ were taken to indicate aberrant p53 expression for IHC results, irrespective of the classification used in the original papers. This was because the majority of papers used this system. Thirdly, in studies using more than one method to evaluate p53 dysfunction, ‘aberrant p53’ refers to the proportion of samples with aberrant p53 by any technique used in the study, that is, samples showing aberrant p53 with one technique, but not all techniques, were still counted as aberrant. While such assumptions may be controversial, they were applied equally to all studies and to both HPV-positive and HPV-negative samples (where HPV testing was used), thus minimising bias. In a rare condition such as VSCC, combining a large number of small series can reveal important trends, which might otherwise be missed.

### Statistical analysis

Proportions of samples showing p53 immunoreactivity and LOH were compared using Fisher's exact test or the *χ*^2^ test, where appropriate. In order to take into account the differing proportions of noninformative cases in the different sample groups, we calculated the fractional regional allelic loss (FRL) for each sample (Wistube *et al*, 1997). FRL for each sample=total number of loci undergoing LOH/total number of informative loci. FRL scores for sample groups were compared using the nonparametric Wilcoxon test. Meta-analysis of pooled results from previous published series was performed using the *χ*^2^ test. Significance was taken at the 5% level.

## RESULTS

The median age of patients with HPV-positive VSCC was 67 years (range 21–94 years) compared with 77 years in the HPV-negative VSCC patients (range 49–91 years). Only four cases (all HPV-positive VSCC) exhibited >50% of nuclei staining positive with DO7. These cases were combined with the samples exhibiting 10–50% of nuclei staining positive with DO7 to provide the ‘p53-positive’ group.

The results of p53 staining in the different VIN and VSCC sample groups are shown in Table 1

**Table 1:** p53 immunoreactivity in VSCC and VIN

	**p53 immunoreactivity**	
**Sample type**	**Positive *n* (%)**	**Negative *n* (%)**
HPV-negative VSCC^a^	4 (31)	9 (69)
HPV-positive VSCC^a^,^b^	12 (22)	42 (78)
VIN associated with VSCC^b^	3 (14)	18 (86)
Lone VIN I, II, III^b^	1 (20), 0 (0), 0 (0)	4 (80), 4 (1 0 0), 43 (1 0 0)

a Fisher's exact test n.s. (*P*<0.74).

b *χ*^2^=10.71, *P*<0.005.

. Results for LOH at the p53 locus have been published elsewhere (Rosenthal *et al*, 2001). The combined IHC and LOH results are shown in Table 2

**Table 2:** Aberrant p53 (by LOH and/or immunoreactivity) in VSCC and VIN

	**Aberrant p53 by LOH and/or immunoreactivity**	
**Sample type**	**Positive *n* (%)**	**Negative *n* (%)**
HPV-negative VSCC^a^	5 (38)	8 (62)
HPV-positive VSCC^a^	18 (33)	36 (66)
VIN associated with VSCC^b^	5 (24)	16 (76)
Lone VIN I, II, III^b^	1 (20), 0 (0), 5 (12)	4 (80), 4 (1 0 0), 38 (88)

a Fisher's exact test n.s. (*P*<0.76).

b *χ*^2^=6.23, *P*<0.05.

. There was no significant difference in the proportions of HPV-positive and HPV-negative VSCC samples demonstrating aberrant p53 by immunoreactivity either alone (Table 1) or in combination with LOH (Table 2). There was a significant trend in increasing aberrant p53 by immunoreactivity either alone (Table 1) or in combination with LOH (Table 2), going from lone VIN to VIN associated with VSCC to HPV-positive VSCC. There was a significant correlation between p53 immunoreactivity and LOH at p53 in VSCC and VIN (Table 3

**Table 3:** Correlation between p53 immunoreactivity and LOH at p53 in VSCC and VIN

	**p53 immunoreactivity**	
**LOH at p53**	**Positive**	**Negative**
Loss^a^	9	14
Retention^a^	8	60

a *χ*^2^=8.473, *P*<0.004.

Note that only 91 out of 140 samples from the IHC results can be included in this analysis because eight samples lacked normal tissue for comparison with neoplastic tissue and 41 samples from the LOH analysis were noninformative.

).

**Figure 1:**
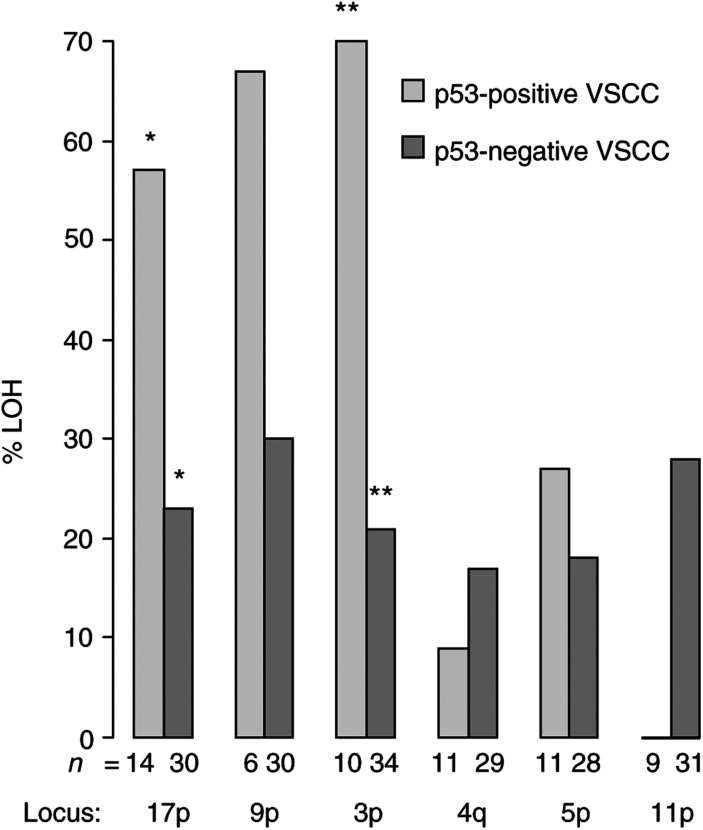
Proportions of p53-positive and p53-negative samples undergoing LOH at six different chromosomal loci. ^*^P<0.028, ^**^P<0.007.

p53 immunoreactivity was significantly associated with overall rates of LOH (median FRL 0.40 *vs* 0.25, *P*<0.027 in p53-positive VSCC *vs* p53-negative VSCC). Figure 1

shows the proportions of p53-positive and p53-negative samples undergoing LOH at six different chromosomal loci. LOH at 17p13 (p53 locus), 9p21 (p16 locus) and 3p25 was more common in p53-positive VSCC compared with p53-negative VSCC, but this only reached statistical significance at 17p13 and 3p25 (57 *vs* 23%, *P*<0.028 and 70 *vs* 21%, *P*<0.007, respectively).

The meta-analyses of previous studies of p53 in VSCC and VIN, along with our own results, are shown in Table 4

**Table 4:** Studies of p53 in VSCC

**Study**	**No. of VSCC samples**	**Technique for HPV/p53 detection**	**Proportion of HPV-positive VSCC with aberrant p53 (%)**	**Proportion of HPV-negative VSCC with aberrant p53 (%)**
Tervahauta *et al* (1993), Kurvinen *et al* (1993)	6	CP+RE+ISH/IHC+SSCP+Seq	2/5 (40)	1/1 (1 0 0)
Lee *et al* (1994)	21	SB+CP+TSP/SSCP +Seq	1/12 (8)	4/9 (44)
Pilotti *et al* (1995)	23	ISH+SB/IHC+SSCP+Seq	4/11 (36)	8/12 (75)
Milde-Langosch *et al* (1995)	40	CP+TSP/IHC+TGGE+Seq	5/11 (46)	19/29 (66)
Kim *et al* (1996)	18	CP+Seq+SB/Seq	0/7 (0)	4/11 (36)
Kagie *et al* (1997b)	66	CP+Seq/IHC	4/12 (33)	31/54 (58)
Pinto *et al* (1999)	12^a^	CP+RFLP+TSP/LOH	3/7 (43)	4/5 (80)
Flowers *et al* (1999)	29^b^	CP+TSP/LOH+SSCP+Seq	4/14 (29)	9/15 (60)
Ngan *et al* (1999)	48	CP+TSP+SB/IHC+SSCP+Seq	11/23 (48)	16/25 (64)
Present study	67	CP+Seq/LOH+IHC	18/54 (33)	5/13 (38)
Total	330	–	52/156 (33)*	101/174 (58)*

a A total of 16 cases, but four are noninformative.

b In all, 30 cases, but one case is both noninformative and with no mutation.

* *P*<0.0001.

CP=consensus primers, IHC=immunohistochemistry, ISH=*in situ* hybridisation, RE=restriction enzyme digestion, RFLP=restriction fragment length polymorphism, Seq=DNA sequencing, SB=Southern blotting, SSCP=single stranded conformational polymorphism analysis, TSP=type-specific primers, TGGE=temperature gel gradient electrophoresis.

and

**5:** Studies of p53 in VIN

**Study**	**No. and type of VIN samples**	**Technique for HPV/p53 detection**	**Proportion of VIN with aberrant p53 (%)**
Kurvinen *et al* (1993), Tervahauta *et al* (1993)	2 lone VIN	CP+RE+ISH/IHC+SSCP+Seq	0/2 (0)
Pilotti *et al* (1995)	6 lone VIN	ISH+SB/IHC+SSCP+Seq	0/6 (0)
Milde-Langosch *et al* (1995)	12 VIN associated with VSCC	CP+TSP/IHC+TGGE+Seq	4/12 (33)
Kagie *et al* (1997a)	60 VIN associated with VSCC	CP+Seq/IHC	10/60 (17)
McConnell *et al* (1997)	33 VIN associated with VSCC	Not performed/IHC	17/33 (52)
Kohlberger *et al* 1998	28 lone VIN	CP/IHC	0/28 (0)
Emanuels *et al* 1999	14 VIN associated with VSCC	Not performed/IHC	3/14 (21)
Flowers *et al* 1999	20 VIN associated with VSCC	CP+TSP/LOH+SSCP+Seq	1/20 (5)
Present study	21 VIN associated with VSCC, 52 lone VIN	CP+Seq/LOH+IHC	5/21 (24) VIN associated with VSCC 6/52 (12) lone VIN
			
Total for VIN not associated with VSCC (lone VIN)	88	–	6/88 (7)*
Total for VIN associated with VSCC	160	–	40/160 (25)*

* *P*<0.0004.

, respectively. Overall, a significantly higher proportion of HPV-negative VSCC samples demonstrated aberrant p53 by one or more methods used, compared with HPV-positive VSCC samples (Table 4, 33 *vs* 58%, respectively, *P*<0.0001). Overall, there was a significantly higher proportion of aberrant p53 in VIN associated with VSCC compared with lone VIN (Table 5, 25 *vs* 7%, respectively, *P*<0.0004).

## DISCUSSION

We set out to compare the rates of p53 disruption in VIN and HPV-positive and HPV-negative VSCC in an attempt to establish whether p53 might be involved in progression from VIN to VSCC, and whether vulval carcinogenesis in the absence of HPV infection requires disruption of p53. p53 has been extensively studied in vulval carcinogenesis. We are aware of 11 studies that have examined p53 disruption in HPV-typed vulval neoplasia (Kurvinen *et al*, 1993; Tervahauta *et al*, 1993; Lee *et al*, 1994; Milde-Langosch *et al*, 1995; Pilotti *et al*, 1995; Kim *et al*, 1996; Kagie *et al*, 1997a,1997b; Kohlberger *et al*, 1998; Flowers *et al*, 1999; Ngan *et al*, 1999). A further four studies (McConnell *et al*, 1997; Sliutz *et al*, 1997; Emanuels *et al*, 1999; Scheistrøen *et al*, 1999) failed to examine HPV status, but two of these included VIN samples (McConnell *et al*, 1997; Emanuels *et al*, 1999). Combining the data from these studies and our own, it appears that VIN associated with VSCC is significantly more likely to demonstrate aberrant p53 function than lone VIN (Table 5, 25 *vs* 7%, *P*<0.0004), suggesting that p53 could be a marker to identify women at risk of progression from VIN to VSCC. In addition, p53 immunoreactivity is only ever present in VIN when it is associated with cancer. Even the single case of lone VIN I, which demonstrated abnormal p53 immunoreactivity in our study, came from a women who had invasive VSCC 4 years previously. However, only approximately 25% of VIN associated with VSCC demonstrated aberrant p53 function in the meta-analysis. While this proportion is too low to predict which women with VIN will develop VSCC, it does suggest that any women with VIN staining positive for p53 should be carefully assessed to exclude invasion.

In our series, aberrant p53 (by IHC or LOH and IHC combined) was more frequent in HPV-positive VSCC than in the associated VIN, which in turn had more aberrant p53 than lone VIN, suggesting that p53 might be involved in progression to invasive disease. This is surprising when one considers the probable aetiology of VIN. In cervical neoplasia, HPV E6 and E7 oncoproteins inhibit cell cycle arrest and apoptosis, allowing the proliferation of mutant clones. The almost universal presence of oncogenic HPV types in VIN and VIN-associated VSCC (Beckmann *et al*, 1991; Hording *et al*, 1995; Van Beurden *et al*, 1995; Rosenthal *et al*, 2001), along with the fact that HPV infection is associated with transcription of E6 and E7 in VIN III (Higgins *et al*, 1991; Jochmus *et al*, 1993; Van Beurden *et al*, 1995) and VSCC (Higgins *et al*, 1991; Park *et al*, 1991), suggests that HPV can also induce some cases of VSCC in a manner analogous to its effects in the cervix. If oncogenic HPV precipitates the accumulation of mutations throughout the genome in vulval neoplasia, then p53 mutations may occur. As mutant p53 has a longer half-life than wild type, allowing its recognition by the DO7 antibody, it may be that p53 immunoreactivity in VIN and VSCC may simply be an index of the mutation burden of the tissue. This would also explain our finding of nearly double the FRL in p53-positive VSCC compared with p53-negative VSCC (median FRL 0.40 *vs* 0.25, respectively, *P*<0.027).

We found a significant association between LOH at p53 and p53 immunoreactivity (Table 3, *P*<0.004). However, despite this correlation, LOH at p53 frequently occurred in the absence of p53 immunoreactivity. This is not surprising, as LOH does not prove that the retained allele is mutated. p53-positive VSCC underwent LOH at 3p25 significantly more often than p53-negative VSCC (Figure 1, 70 *vs* 21%, *P*<0.007), suggesting that LOH at this locus might act synergistically with p53 dysfunction in the carcinogenic pathway. Interestingly, the Fanconi anaemia complementation group D gene has recently been localised to 3p25.3 (Hejna *et al*, 2000) and VSCC has been reported in patients with Fanconi anaemia at ages much younger than is typical of VSCC (Arnold *et al*, 1980; Kennedy and Hart, 1982; Wilkinson *et al*, 1984).

Meta-analysis reveals that HPV-negative VSCC appears to undergo a significantly increased frequency of p53 disruption compared with HPV-positive VSCC (Table 4, 58 *vs* 33%, *P*<0.0001), indicating that p53 is involved in the majority of HPV-negative VSCC. However, our own study and meta-analysis support the view that p53 disruption is not obligatory in HPV-independent pathways of vulval carcinogenesis.

In conclusion, we found that p53 immunoreactivity in VIN is associated with the presence of invasive disease, implying that any case of VIN staining positive for p53 should be checked carefully for the presence of occult invasion. Meta-analysis suggests that HPV-negative VSCC has a significantly greater proportion of aberrant p53 compared to HPV-positive VSCC; however, nearly half of all HPV-negative VSCC has no evidence of p53 dysfunction, thus implicating other molecular events in this pathway. We observed a significant association between p53 immunoreactivity and LOH at 3p25 suggesting that a tumour suppressor at this locus might act synergistically with p53 dysfunction. p53 immuno-reactivity was also significantly associated with FRL, implicating it as an index of the mutation burden of the tissue.
